# Supplementary material for: Altered Ocular Surface Health Status and Tear Film Immune Profile Due to Prolonged Daily Mask Wear in Health Care Workers
Source: Biomedicines. 2022 May 18;10(5):1160. doi: 10.3390/biomedicines10051160 (PMC9139140; doi:10.3390/biomedicines10051160)
Supplement: Supplementary file 1 [file biomedicines-10-01160-s001.zip › biomedicines-1651854-supplementary.pdf]

Research article

Altered ocular surface health status and tear film immune profile due to prolonged daily mask wear in health care workers

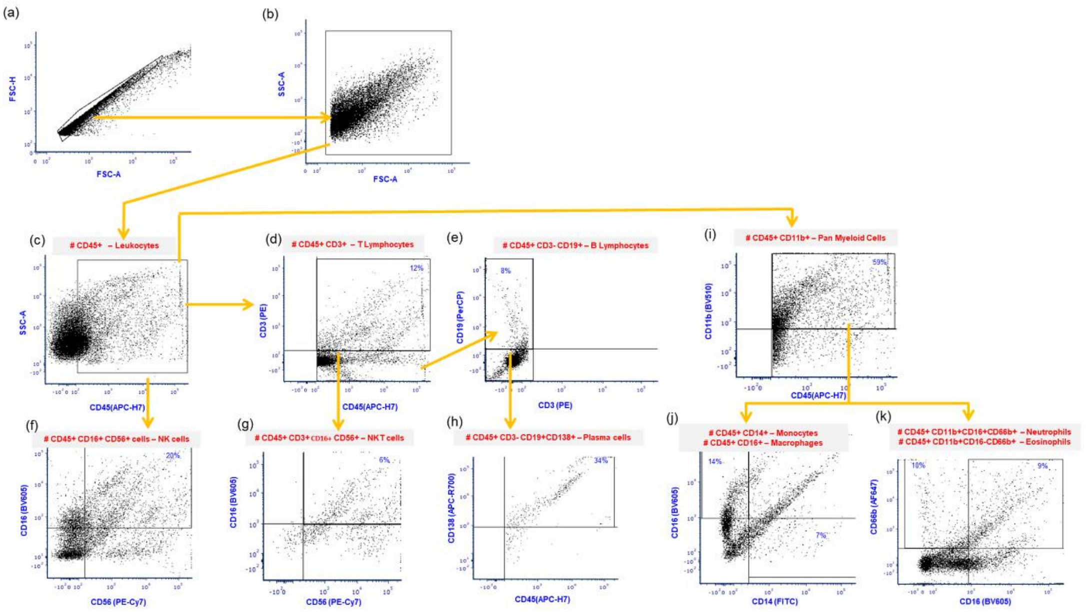

Figure S1: Gating strategy to phenotype ocular surface immune cell subsets: Representative images shows gating strategy used to determine the immune cell populations (specifically Leukocytes, T cells, B cells, Natural Killer cells, NKT cells, Pan Myeloid cells, Monocytes, Macrophages, Neutrophils, Eosinophils) in the ocular surface wash samples. The respective samples were stained for immune cell type-specific fluorochrome-conjugated antibodies and manual gating strategy (marked with arrows) were performed. (a) The scatter plot marked Forward scatter-A (FSC-A) versus Forward scatter-H (FSC-H) represents singlet population and (b) marked region in Forward scatter (FSC-A) versus Side Scatter (SSC-A) plot represents the total cell population. (c) The marked region CD45 (APC-H7) versus Side Scatter (SSC) represents the leukocytes population. (d) The upper right quadrant in this panel indicates T cells positively stained for both CD3 (PE) and CD45 (APC-H7). Bottom right quadrant which indicates leukocytes devoid of T cells were used to determine B cell population in the next panel. (e) The upper left quadrant in the panel represents B cell population - CD19 (PerCP) positive but CD3 (PE) negative cells. (f) The upper right quadrant in this panel indicates CD45 (APC-H7) positive cells stained for both CD16 (BV605) and CD56 (PE-Cy7) and they represent NK cells while (g) NKT cells represented in the plot are T cells shown in panel d that are positively stained for CD16 (BV605) and CD56 (PE-Cy7) as can be seen in the upper right quadrant. (h) plasma cells represented in the plot are B cells shown in panel e that are positively stained for CD138 (APC-R700) as can be seen in the upper right quadrant. (i) The upper right quadrant represents pan-myeloid cells stained positive for both CD45 (APC-H7) and CD11b (BV510). (j) The scatter plot represents pan-myeloid cells from panel h that are positive cells stained for CD14 (FITC) or CD16(BV605). The lower right quadrant shows monocytes that are exclusively positive for CD14 (FITC) while upper left quadrant shows macrophages that are exclusively stained positive for CD16 (BV605). (k) CD16 (BV605) versus CD66b (AF647) plot is used to identify neutrophils and eosinophils within in the pan-myeloid cells as seen in panel i. Neutrophils are cells that are positive for both CD16 (BV605) and CD66b (AF647) – upper right quadrant. Eosinophils are represented in the upper left quadrant are stained only for CD66b (AF647). The percentage of each immune cell subsets for each study subject was computed using number of positively stained cells (cell specific markers) to number of CD45 positive cells (leukocytes) obtained from FCS express 6.

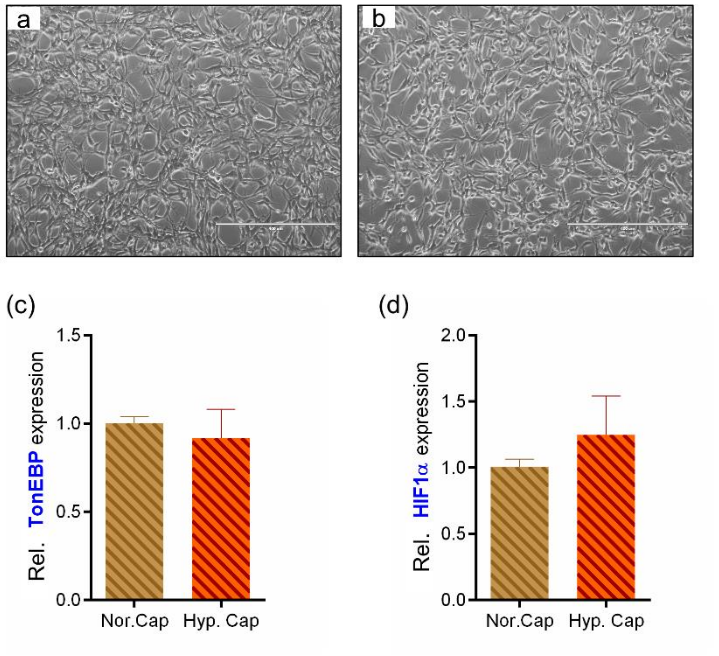

Figure S2: Effect of hypercapnia on cellular morphology and osmotic stress response genes in human corneal epithelial cells. Panels shows the morphology of SV40 immortalized human corneal epithelial cells (HCE2) following exposure to either (a) 5% CO2 (Normocapnia – Nor. Cap) or (b) 20% CO2 (Hypercapnia – Hyp. Cap), in vitro for a period of 24 hours in vitro using a bright field microscope at 10x magnification. The microscopic images shown are representative images of three different fields from three independent experiments. Graphs indicate mean relative mRNA expression of TonEBP (c) and HIF1α (b) in SV40 immortalized human corneal epithelial cells (HCE2) exposed to either 5% CO2 (Normocapnia – Nor. Cap) or 20% CO2 (Hypercapnia – Hyp. Cap), in vitro for a period of 24 hours. The expression of TonEBP and HIF1α were normalized to expression of β-Actin (housekeeping gene). Bar graph indicates Mean±SEM from two technical replicates for each of the three biological replicate experiments. TonEBP – Tonicity-responsive enhancer-binding protein; HIF1α – Hypoxia-inducible factor-1α.

Table S1: The levels of cytokines in the tear fluid of study subjects.

Analytes (pg/ml)	Pre-FM			Post-FM			P value
	**Mean**	**Stdev**	**SEM**	**Mean**	**Stdev**	**SEM**	
IL-1α	56	61	10	81	58	10	0.037
IL-1β	166	171	29	296	196	34	0.005
IL-2	15	32	5	44	85	15	0.033
IL-6	4	8	1	2	8	1	0.045
IL-8	1189	3962	679	50	45	8	< 0.0001
IL-12/IL-23p40	1297	1853	318	542	725	124	0.095
IL-13	14	27	5	3	10	2	0.030
IL-17A	1	3	1	1	4	1	0.882
IL-18	95	102	18	41	60	10	0.001
IL-21	602	933	160	1099	1761	302	0.249
IL-33	522	960	165	1322	1053	181	0.001
IFNα	15	24	4	26	66	11	0.723
IFNβ	525	602	103	902	720	123	0.010
IFNγ	2	11	2	8	33	6	0.465
TNFα	5	10	2	1	2	0	0.101

Table S2: The levels of chemokines and growth factors in the tear fluid of study subjects.

Analytes (pg/ml)	Pre-FM			Post-FM			P value
	Mean	Stdev	SEM	Mean	Stdev	SEM	
Fractalkine	1228	1146	197	937	962	165	0.488
GROα	1950	1988	341	1489	2099	360	0.661
IP-10	688731	3967968	680501	9091	18000	3087	0.278
I-TAC	997	2426	416	153	145	25	0.002
MCP-1	182	164	28	187	172	30	0.919
MIG	768	2026	353	564	1543	265	0.129
RANTES	57	105	18	16	19	3	0.001
BDNF	169	197	34	350	246	42	0.001
NGF	10	7	1	15	15	3	0.062
HGF	1708	1247	214	1157	1252	215	0.005
VEGF	721	529	91	358	251	43	< 0.0001

Table S3: The levels of soluble cell adhesion molecules, soluble receptors and enzymes in the tear fluid of study subjects.

Analytes (pg/ml)	Pre-FM			Post-FM			P value
	Mean	Stdev	SEM	Mean	Stdev	SEM	
sICAM1	6244	10808	1854	2864	5146	882	0.062
sVCAM	440	781	134	42	126	22	0.001
sL-selectin	2249	4114	706	130	449	77	0.001
sP-selectin	710	1635	280	255	517	89	0.184
sIL-1R1	324	264	45	164	270	46	0.001
sIL-1R2	337	620	106	48	39	7	0.024
sIL-2Ra	124	107	18	166	136	23	0.048
sTNFRI	126	153	26	257	311	53	0.001
sTNFRII	44	56	10	14	19	3	0.008
LIF	2268	2146	368	3973	2454	421	0.001
Angiogenin	51572	65803	11285	46033	37368	6409	0.723
NGAL	2319	1936	332	633	549	94	< 0.0001
Granzyme	2811	1718	295	1398	1145	196	0.001
Perforins	409	540	93	618	606	104	0.027
TSLP	102	152	26	264	191	33	0.001
